# Oxidizing EuAl_2_: A Precursor for the Synthesis of Di- and Trivalent Europium Oxides

**DOI:** 10.1021/acs.inorgchem.6c02830

**Published:** 2026-07-31

**Authors:** Cedric Kloos, Elias C. J. Gießelmann, Aaron Haben, Lena Ruck, Jasper A. Baldauf, Joshua Wiethölter, Rainer Pöttgen, Guido Kickelbick, Oliver Janka

**Affiliations:** † Saarland University, Solid State Inorganic Chemistry, Campus C4 1, Saarbrücken 66123, Germany; ‡ Saarland University, WASTe-Elemental Analysis Group, Campus C4 1, Saarbrücken 66123, Germany; § Saarland University, Core Facility ELMAS-CF, Campus C4 1, Saarbrücken 66123, Germany; ∥ 271671 Universität Münster, Institut für Anorganische und Analytische Chemie, Corrensstraße 30, Münster 48149, Germany

## Abstract

Synthesis of europium aluminates is usually conducted from the binary oxides (EuO/Eu_2_O_3_ + Al_2_O_3_) at high temperatures alongside a reductive gas for the stabilization of Eu^2+^. We are reporting on an alternative pathway starting from EuAl_2_ (cubic Laves phase, MgCu_2_ type, *Fd*3̅*m*), which was oxidized under different conditions. By heating the intermetallic precursor to different temperatures, first amorphous and at higher temperatures highly crystalline products were observed. However, the question of the Eu valence state in the product will immediately arise. Depending on the reaction procedure used both, the di- and trivalent state could be achieved in the reaction with elemental oxygen. Eu^III^AlO_3_ (GdFeO_3_ type, *Pnma*) was observed as the sole crystalline product with excess O_2_ in the powder X-ray diffraction experiments. The differing chemical composition could be explained by the formation of amorphous γ-Al_2_O_3_ proven by ^27^Al solid-state NMR spectroscopy. Oxidizing EuAl_2_ under controlled conditions yields X-ray pure Eu^II^Al_2_O_4_ (SrAl_2_O_4_ type, *P*2_1_). Surprisingly, annealing of the Eu^III^AlO_3_/amorphous γ-Al_2_O_3_ mixture under vacuum led to the formation of Eu^II^Al_2_O_4_, which was investigated in detail. The different oxidation states of the Eu atoms were proven by magnetic measurements, ^151^Eu Mössbauer spectroscopic investigations, and photoluminescence studies.

## Graphical abstract

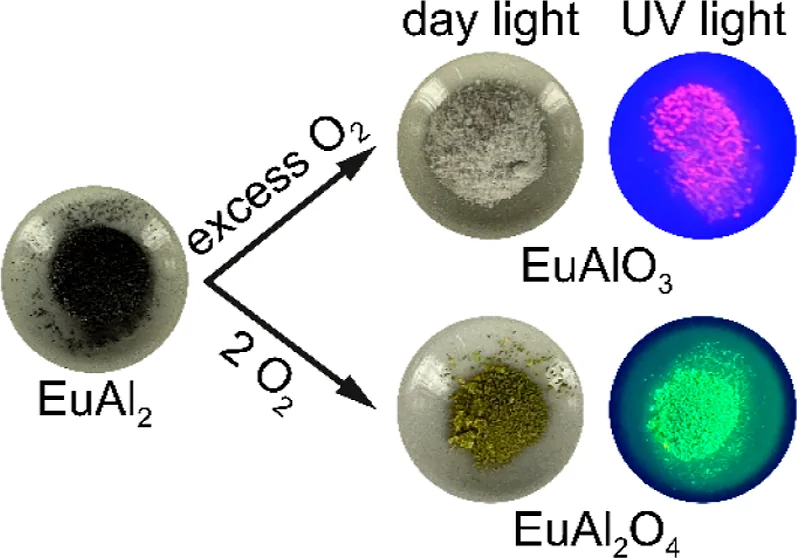

## 1 Introduction

The rare earth elements (*RE*, electron configuration [Xe] 6s^2^ 5d^1^ 4f^*n*^) typically adopt a stable trivalent oxidation state as cations (electron configuration [Xe] 4f^*n*^) due to the shielded character of the f-orbitals. For some of the rare earth elements, however, other oxidation states are rather easily accessible. Cerium ([Xe] 6s^2^ 5d^1^ 4f^1^) for example can be observed as formally tri- and tetravalent cation in, e.g., Ce_2_O_3_ (*A*-La_2_O_3_ type, space group *P*321)^1^ and CeO_2_ (CaF_2_ type, space group *Fm*3̅*m*)^2^ due to the emptied f-shell. Ytterbium ([Xe] 6s^2^ 5d^1^ 4f^13^) in contrast can form di- ([Xe] 4f^14^) and trivalent ([Xe] 4f^13^) cations with Yb^2+^ having a filled f-shell. Europium ([Xe] 6s^2^ 5d^1^ 4f^6^), finally, also exhibits di- ([Xe] 4f^7^) and trivalent ([Xe] 4f^6^) cations with the Eu^2+^ cations stabilized by the half-filled f-shell. This leads to several interesting consequences. Elemental europium for example is a so-called two electron metal.^3–5^ Also, Eu^2+^ is significantly larger (*r*_ion_ = 117 pm for CN = 6) compared to Eu^3+^ (*r*_ion_ = 95 pm for CN = 6)^6,7^ often making Eu^2+^ and Sr^2+^ (*r*_ion_ = 118 pm for CN = 6 ^6,7^ ) crystal chemical analogues. The effective magnetic moment of Eu^2+^ is equal to that of Gd^3+^ (both μ_eff_ = 7.94 μ_B_ ^8,9^ ), while Eu^3+^ ([Xe] 4f^6^; *S* = 3, *L* = 3) is a so-called van Vleck ion exhibiting a ^7^F_0_ ground state with the first excited state (^7^F_1_) being accessible at room temperature.^8–11^ Also the optical properties are significantly different. Due to the shielding of the 4f orbitals by the filled 5s and 5p orbitals, the influence of the host structure onto the optical transitions in the Eu^3+^ cation is small and furthermore, optical transitions are forbidden due to the parity selection rule. This leads to a sharp emission line around 620 nm.^12,13^ Eu^2+^ in contrast exhibits allowed f→d transitions, leading to broad absorption and emission bands and a significant influence of the host structure onto the emission.

In intermetallic compounds, the predominant oxidation state of Eu is the divalent one,^14^ while trivalent compounds are scarce with below ∼20 reported binary and ternary compounds.^15^ In addition to the two classical oxidation states, also static mixed valence (EuPtP^16^), valence fluctuations (EuCu_2_Si_2_ ^17,18^ ) or pressure and temperature-dependent valence phase transitions (Eu­(Ir_1–*x*_Pd_*x*_)_2_Si_2_,^19^ Eu(Pd_1–*x*_Au_*x*_)_2_Si_2_,^20^ Eu_2_Pt_6_Al_15_ and Eu_2_(Pt_1–*x*_ *T*_*x*_)_6_Al_15_ ^21–24^ ) occur. But also, in the chalcogenides, both oxidation states (e.g., EuO^25^ and Eu_2_O_3_ ^26^ or EuS^27^ and Eu_2_S_3_ ^28^) along with a static mixed valence compound (Eu_3_O_4_ ^29–31^ or Eu_3_S_4_ ^32^) are observed.

Recently, we have shown that the intermetallic compounds CaAl_2_ can be oxidized by elemental oxygen with a complex reaction, leading to the formation of mayenite type Ca_12_Al_14_O_33_ as the main product and only repeated annealing lead to the formation of the anticipated product CaAl_2_O_4_.^33^ Activation of the precursor by ball-milling could significantly shift the product spectrum toward CaAl_2_O_4_.^34^ ScAl_2_ in contrast does not form ternary oxides.^35^ Interestingly, CaAl_2_ and SrAl_2_ can be oxidized with elemental sulfur leading to phase pure products of CaAl_2_S_4_ and SrAl_2_S_4_.^36^ This reaction is also possible with EuAl_2_ leading to phase pure samples of EuAl_2_S_4_, which show a bright green luminescence indicative of divalent Eu.^37^ Interestingly, only Eu^2+^ is observed as shown by magnetization experiments and ^151^Eu Mössbauer spectroscopic measurements, in contrast to the possibility of the formation of Eu^3+^ as shown in other sulfides (*vide supra*).

This led to the outright question of what happens during the oxidation of SrAl_2_ and EuAl_2_ in the presence of elemental O_2_. For SrAl_2_, the formation of the corresponding monoclinic oxide SrAl_2_O_4_ ^38^ can be hypothesized, however, the formation of other oxides, like in the case of CaAl_2_,^33^ is possible. For EuAl_2_, not only different oxides by composition have been reported but also different oxidation states of the Eu atoms within these compounds were observed. EuAl_2_O_4_ contains Eu^2+^ and crystallizes in the monoclinic SrAl_2_O_4_ type structure,^39^ highlighting once again the similarities of Sr^2+^ and Eu^2+^, but also oxides with trivalent europium, like the perovskite-related EuAlO_3_ (GdFeO_3_ type)^40^ or the garnet-type Eu_3_Al_5_O_12_,^41^ have been reported.

Here, we illustrate the differences in the oxidation behavior of intermetallic SrAl_2_ and EuAl_2_, the possibility to control the Eu oxidation state, the structural characterization alongside element analytic techniques, and ^27^Al solid-state NMR spectroscopic studies. The optical properties have been investigated by photoluminescence spectroscopy; the oxidation state of the Eu atoms has been confirmed by magnetization and ^151^Eu Mössbauer spectroscopic measurements. This illustrates the potential of this method to systematically investigate structure–property relationships with respect to the physical properties of these compounds.

## 2 Experimental Section

### 2.1 Synthesis

Starting materials for the synthesis of SrAl_2_ and EuAl_2_ were strontium, europium, and aluminum pieces, purchased from Onyxmet (Olsztyn, Poland), all with stated purities >99.9%. The Sr, Eu, and Al pieces were mechanically cut into smaller pieces. The elements were mixed in the ideal stoichiometric ratios with the Sr or Eu pieces wrapped in aluminum foil (Alujet, Mammendorf, Germany) or reacted in a sealed Ta-ampule in an induction furnace under purified argon.^42^ The argon gas (5.0, Air Liquide, Düsseldorf, Germany) was purified over molecular sieves, silica gel, and a titanium sponge (873 K).

For the oxidation in the STA (simultaneous thermal analyses) system, we refer to Section 2.3 (*vide infra*). Bulk oxidation reactions were performed in a custom-built tube furnace (Carbolite Gero GmbH & Co. KG, Neuhausen, Germany) setup. The furnace was operated under pure O_2_ (5.0, Air Liquide, Düsseldorf, Germany). The samples were placed in porcelain boats and centered in the tube furnace. The oven was purged with purified argon (titanium sponge at 873 K, mole sieve, P_2_O_5_) for 1 h prior to the reaction with O_2_ to avoid a potential reaction with N_2_. The flow rate of O_2_ was regulated using variable area flow meters (Kobold Messring GmbH, Hofheim am Taunus, Germany). For the oxidation reactions, pure oxygen with a flow rate of 20 mL min^–1^ was used. The oxidation reactions were conducted at 773, 1073, and 1273 K (heating rate of 200 K h^–1^) followed by direct cooling or annealing for 5 h.

BaO_2_ was purchased from abcr (Karlsruhe, Germany) with a stated purity of 99.9%. To ensure a stoichiometric oxygen release from BaO_2_, STA measurements were performed beforehand underlining the correct composition. A ceramic crucible containing 1 equiv of EuAl_2_ was placed above a second crucible filled with 3–4 equiv of BaO_2_ inside a fused silica tube (13 mm inner diameter, wall thickness 1 mm). The silica tube was dried at 373 K beforehand, sealed under vacuum, and positioned vertically in a muffle furnace. The furnace was heated to 1073 K at a rate of 200 K h^–1^, held at this temperature for 48 h, and then cooled to room temperature.

### 2.2 Powder X-ray Diffraction

The pulverized samples of all discussed compounds were investigated by powder X-ray diffraction experiments at room temperature on a D8-A25-Advance diffractometer (Bruker AXS, Karlsruhe, Germany) in Bragg–Brentano θ-θ-geometry (goniometer radius 280 mm) with nonmonochromatic Cu *K*_α1,2_-radiation (λ = 154.0596 and 154.4425 pm). Diffraction patterns were recorded between 6° and 130° 2θ with a step size of 0.013° and a total scan time of 1 h. A 12 μm Ni foil working as *K*_β_ filter and a variable divergence slit were mounted at the primary beam side. On the secondary beam side, a LYNXEYE detector with 192 channels was used. The recorded data was evaluated using Bruker TOPAS 5.0 software^43^ employing the fundamental parameter approach and the Rietveld method.^44,45^ Table S1 lists the refined lattice parameters of the starting materials and the main oxidation products. The unit cell parameters and atomic positions used for the Rietveld refinements were taken from the Pearson database.^46^

### 2.3 Thermal Analysis

Simultaneous thermogravimetric analysis (TGA) and differential scanning calorimetry (DSC) were carried out with a TGA/DSC 1 Star HT/1600 system (Mettler Toledo, Columbus, OH, USA) under an Ar/O_2_ atmosphere with flow rates of 40 mL min^–1^ each. Temperature programs are given with the experiments, the heating rates were typically 20 K min^–1^ if not specified otherwise. Samples were placed into alumina crucibles (ø = 6 mm, h = 4.5 mm) for the measurements.

### 2.4 Energy-Dispersive X-ray Spectroscopy

The powdered samples of nominal EuAlO_3_ and EuAl_2_O_4_ were semi-quantitatively analyzed on a JEOL 7000F (JEOL, Tokyo, Japan) scanning electron microscope equipped with an EDAX Genesis 2000 EDX detector (EDAX, Unterschleißheim, Germany). The powdered samples were sprinkled on conductive carbon tape and coated with Au. One area scan and three independent data points were measured. The results of the SEM/EDX investigations are given in Table S2.

### 2.5 Inductively Coupled Plasma Mass Spectrometry

Whenever the use of water was necessary, ultrapure water (0.055 μS cm^–1^) (PURELAB Chorus 1 ultrapure water filtration unit, Elga LabWater, High Wycombe, UK) was used. Prior to the ICP-QQQ quantification, approximately 11–13 mg of samples were digested in 8 mL of aqua regia (HCl: 30% Suprapur, Merck Supelco, Darmstadt, Germany; HNO_3_: Rotipuran Supra 69%, Carl Roth, Karlsruhe, Germany) using a Multiwave 5001 with SVT50 vessels (Anton Paar, Ostfildern-Scharnhausen, Germany). The digested samples were then transferred quantitatively into volumetric flasks (25 mL PP GL 18, VITLAB, Großostheim, Germany) and filled up to volume with ultrapure water. For the analysis, these solutions were diluted by a factor of 10,000 (via a two-step dilution).

The elemental quantification was conducted with a commercial ICP-QQQ system (8900 Triple Quad and SPS4 autosampler, Agilent, Santa Clara, USA). All measurement solutions were prepared to a total volume of 10 mL and contained 300 μL of HNO_3_ (see above), as well as 10 μg L^–1^ each of the internal standards: Sc (1 g L^–1^ in 5% HNO_3_, Alfa, Karlsruhe, Germany), Y and Ho (1 g L^–1^ in 2–3% HNO_3_, Merck Certipur, Darmstadt, Germany). For quantification purposes, an external calibration was performed (up to 50 μg L^–1^) using Al (1 g L^–1^ in 2–3% HNO_3_, Merck Certipur, Darmstadt, Germany) and Eu (1 g L^–1^ in nitric acid, Merck TraceCERT, Darmstadt, Germany) ICP–MS standard solutions.

Both ^27^Al and ^153^Eu were measured on-mass (Q1/Q2 = 27/27 and Q1/Q2 = 153/153) in the H_2_- and O_2_-reaction gas modes, with ^153^Eu also being measured in mass-shift (Q1/Q2 = 153/169) in the O_2_-reaction gas mode. For data evaluation, the measured values obtained from various measurement modes were averaged.

### 2.6 Physical Property Measurements

The polycrystalline samples of EuAlO_3_ and EuAl_2_O_4_ were investigated by temperature-dependent magnetic susceptibility measurements at an external field of 10 kOe (1 kOe = 7.96 × 10^4^ A m^–1^). The samples were filled into PP capsules and attached to the sample holder rod of a vibrating sample magnetometer (VSM) option of a Dynacool Physical Property Measurement System (PPMS) by Quantum Design (San Diego, USA). The magnetization data *M*(*T*) of the samples were investigated in the temperature range between 2 and 300 K with an applied field of 10 kOe.

### 2.7 ^151^Eu Mössbauer Spectroscopic Investigations

The 21.53 keV transition of ^151^Eu with an activity of 65 MBq (1% of the total activity of a ^151^Sm/EuF_3_ source) was used for the ^151^Eu Mössbauer spectroscopic experiments, which were conducted in the usual transmission geometry. The velocity was calibrated using α-Fe. All measurements were performed with a liquid N_2_ bath cryostat system. The temperature of the absorber was set to 78 K, while the source was kept at room temperature. The temperature was controlled by a resistance thermometer (∼0.5 K accuracy). The samples were enclosed in a small PMMA containers, the required sample mass was calculated based on the work by Long et al.^47^ Both samples, EuAl_2_O_4_ and EuAlO_3_, were diluted with SiO_2_. Fitting of the spectra was performed with the WinNormos for Igor7 program package.^48^ The obtained fitting parameters are listed in Table S3.

### 2.8 ^27^Al Solid-State Magic-Angle Spinning NMR

^27^Al solid-state MAS NMR spectra were recorded using an Avance III 400 WB (Bruker, Billerica, USA) at 104.35 MHz using magic-angle spinning (MAS). The samples were used as fine powders and mixed with dried sodium chloride if the amount of sample was not enough. The pure or diluted samples were loaded into a cylindrical ZrO_2_ rotor with a diameter of 4 mm and spun at the magic angle at a frequency of 13 kHz. All experiments conducted were single-pulse experiments with a typical pulse length of 0.83 μs (≈30° pulse) and a relaxation delay of 1 s. Resonance shifts were referenced to an aqueous 1 molar AlCl_3_ solution. The NMR spectra were recorded using Bruker Topspin^49^ software; the analysis was performed with the help of Dmfit software.^50^

### 2.9 Fluorescence and UV–vis Spectroscopy

Fluorescence spectroscopy was performed on a FluoroMax 4 Spectrofluorometer (Horiba Scientific, Kyoto, Japan) with an excitation wavelength of 395 nm for EuAlO_3_ and 375 nm for EuAl_2_O_4_. Both compounds were measured as pure solids in silica glass tubes with an inner diameter of 4 mm. UV–vis diffuse reflectance spectra were recorded on a PerkinElmer Lambda 750 spectrometer (PerkinElmer Inc., Shelton, USA) equipped with a 100 mm integration sphere from 290 to 1500 nm with a 2 nm increment and an integration time of 0.2 s. BaSO_4_ was used as a white reference.

## 3 Results

### 3.1 Crystal Chemistry

Before we discuss the results from the oxidation reactions, the basic structural chemistry of the compounds should be briefly described first. SrAl_2_ crystallizes in the orthorhombic KHg_2_-type structure (space group *Imma*, Figure 1a),^51,52^ which is derived from the AlB_2_-type.^53^ The Al atoms form corrugated six-membered rings, the Sr atoms occupy the cavities. EuAl_2_ crystallizes in the MgCu_2_-type structure (cubic Laves, space group *Fd*3̅*m*, Figure 1b)^54^ with the Eu atoms on the Mg site (8*b*) of the prototype and Al occupying the Cu positions (16*c*). More details on the Laves phases can be found in review articles.^55,56^

**Figure 1:**
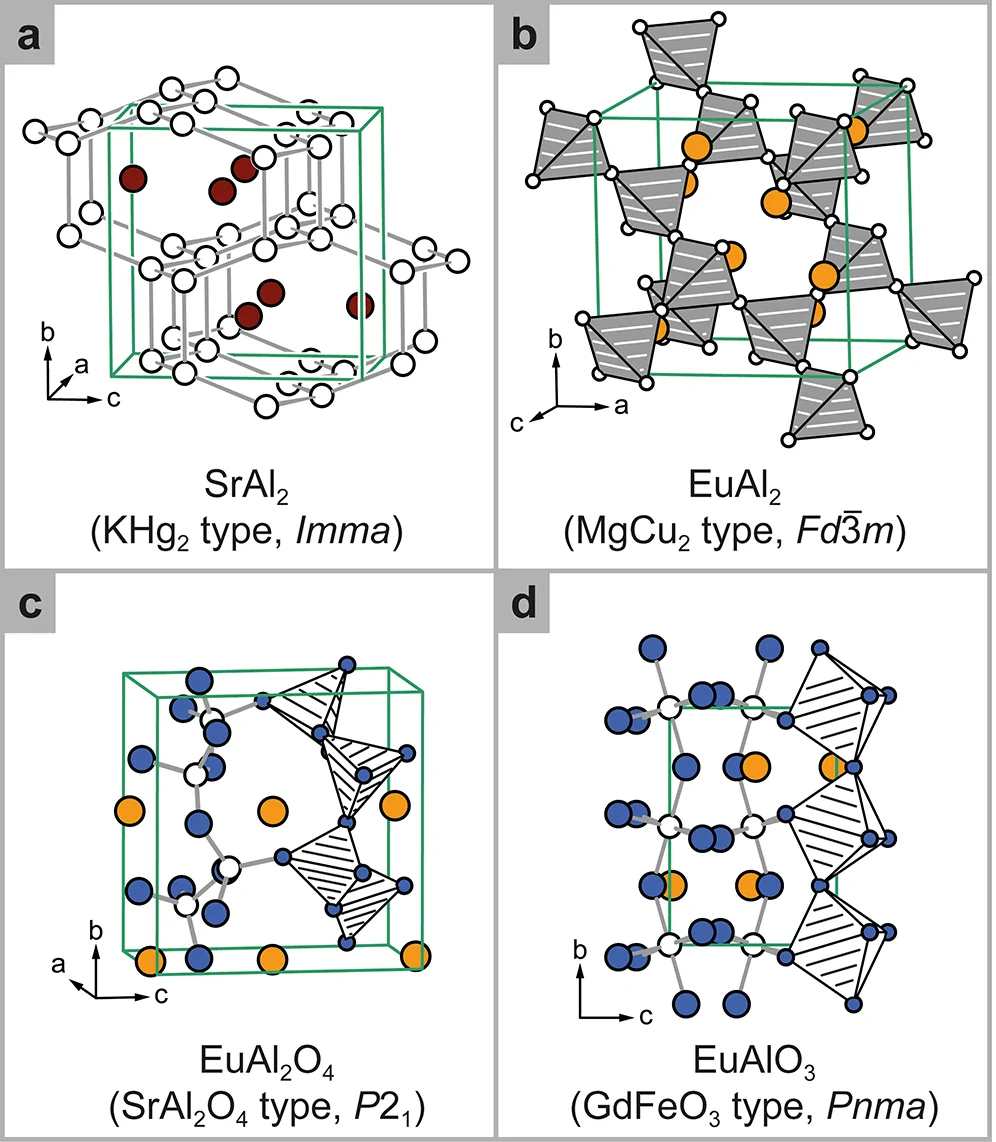
Crystal structures of (a) orthorhombic SrAl_2_ (KHg_2_, *Imma*), (b) cubic EuAl_2_ (MgCu_2_-type, *Fd*3̅*m*), (c) monoclinic Eu^II^Al_2_O_4_ (SrAl_2_O_4_-type, *P*2_1_), and (d) orthorhombic Eu^III^AlO_3_ (GdFeO_3_-type, *Pnma*). Important coordination polyhedra are shown in gray/white. Al atoms are shown in white, Sr atoms in burgundy, Eu in orange, and oxygen atoms in blue.

The main difference between the two observed oxides are the [AlO_*x*_] coordination polyhedra. In SrAl_2_O_4_ and Eu^II^Al_2_O_4_ (SrAl_2_O_4_-type, space group *P*2_1_, Figure 1c), the Al atoms are tetrahedrally coordinated by the oxide anions with all corners linked to other [AlO_4_] tetrahedra (Figure 2a). The two crystallographically independent Eu/Sr atoms (Figure 2b) are coordinated by seven (Eu1) and eight (Eu2) oxide anions and are located in the cavities of the [Al_2_O_4_]^2–^ polyanion. The coordination numbers were confirmed by ECoN calculations,^57,58^ use of the program Polynator^59^ assigned a monocapped trigonal prism to Eu1 and a tetragonal antiprism to Eu2. In Eu^III^AlO_3_ (GdFeO_3_-type, space group *Pnma*, Figure 1d), in contrast, [AlO_6_] octahedra are observed sharing all corners with neighboring octahedra. The Eu atoms reside in bicapped trigonal prisms (Figure 2c). Chardi calculations confirm the formal oxidation states of the Eu atoms in both compounds (Table S4).^57,58^

**Figure 2:**
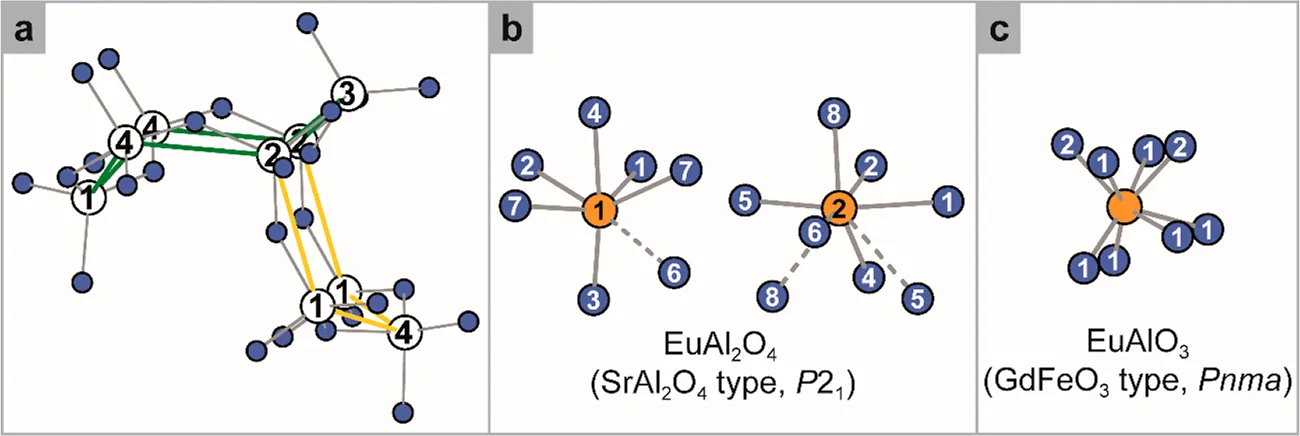
a) Cutout of the [AlO_4_] network of EuAl_2_O_4_. The chair and boat confirmations are highlighted. Coordination environments of the Eu positions in (b) Eu^II^Al_2_O_4_ and (c) Eu^III^AlO_3_. Eu atoms are shown in orange, Al atoms are shown in white, and O atoms are shown as blue spheres.

### 3.2 Oxidation Reactions

The oxidation reactions of SrAl_2_ and EuAl_2_ were subsequently investigated. In the oxidic environment, Sr only exhibits the divalent oxidation state, while Eu can form Eu^2+^ and Eu^3+^. Therefore, one can hypothesize that the oxidation of SrAl_2_ is similar to the one of CaAl_2_, while EuAl_2_ behaves significantly different.

Both SrAl_2_ and EuAl_2_ were prepared from the elements, characterized by powder X-ray diffraction, and subsequently oxidized under different conditions. The powder X-ray diffraction patterns of SrAl_2_ (Figure S1a) and EuAl_2_ (Figure S2a) show that X-ray pure samples with a high crystallite size (>200 nm) could be obtained, the lattice parameters are listed in Table S1.

In the first step, SrAl_2_ and EuAl_2_ were heated under an excess of O_2_ in an STA system. Both samples were heated to 1273 K with 20 K min^–1^ followed by immediate cooling (SrAl_2_) or held for 1 h (EuAl_2_) before cooling to room temperature. In both cases, several exothermic signals are observed upon heating. For SrAl_2_ (Figure 3a), two distinct signals at *T*_1_ = 1006 and *T*_2_ = 1097 K are observed along with a shoulder just below *T*_2_. For EuAl_2_ (Figure 3b) also two exothermic events are observed at temperatures of *T*_1_ = 980 and *T*_2_ = 1170 K. In both cases, also a significant mass increase of 24% for SrAl_2_ and 34% for EuAl_2_ is observed. The theoretical mass increase for the oxidation of SrAl_2_ to SrAl_2_O_4_ is 45%, indicating that no full oxidation has happened. This is in line with the observations made for CaAl_2_.^33^ Here, the oxidation initially leads to the formation of the so-called mayenite (Ca_12_Al_14_O_33_) due to the diffusion of Ca to the surface of the grains followed by the formation of other calcium aluminates and CaAl_2_O_4_. For SrAl_2_ similar results are observed. The initial oxidation leads to the formation of Sr_3_Al_2_O_6_ ^60^ alongside the decomposition of SrAl_2_ into SrAl_4_ ^61^ and elemental Al. The presence of elemental Al can be seen in the cooling curve (Figure 3a) as the exothermic signal at around 925 K corresponds to the melting point of Al. Longer dwelling or repetitive annealing under oxygen leads to an increase in the expected product of monoclinic SrAl_2_O_4_ (Figure S1b), however, after in total 36 h (3 × 12 h) and two intermediate grinding steps, only 87 mass% SrAl_2_O_4_ were observed alongside 9 mass% Sr_3_Al_2_O_6_ and 4 mass% SrAl_12_O_19_ ^62^ according to powder X-ray diffraction studies. The refined lattice parameters are given in Table S1. The observations match the literature data on oxidation reactions of other aluminides.^33,34^ Since only complex mixtures were obtained, the possibility to synthesize phase pure products by this method was not further investigated for this system.

**Figure 3:**
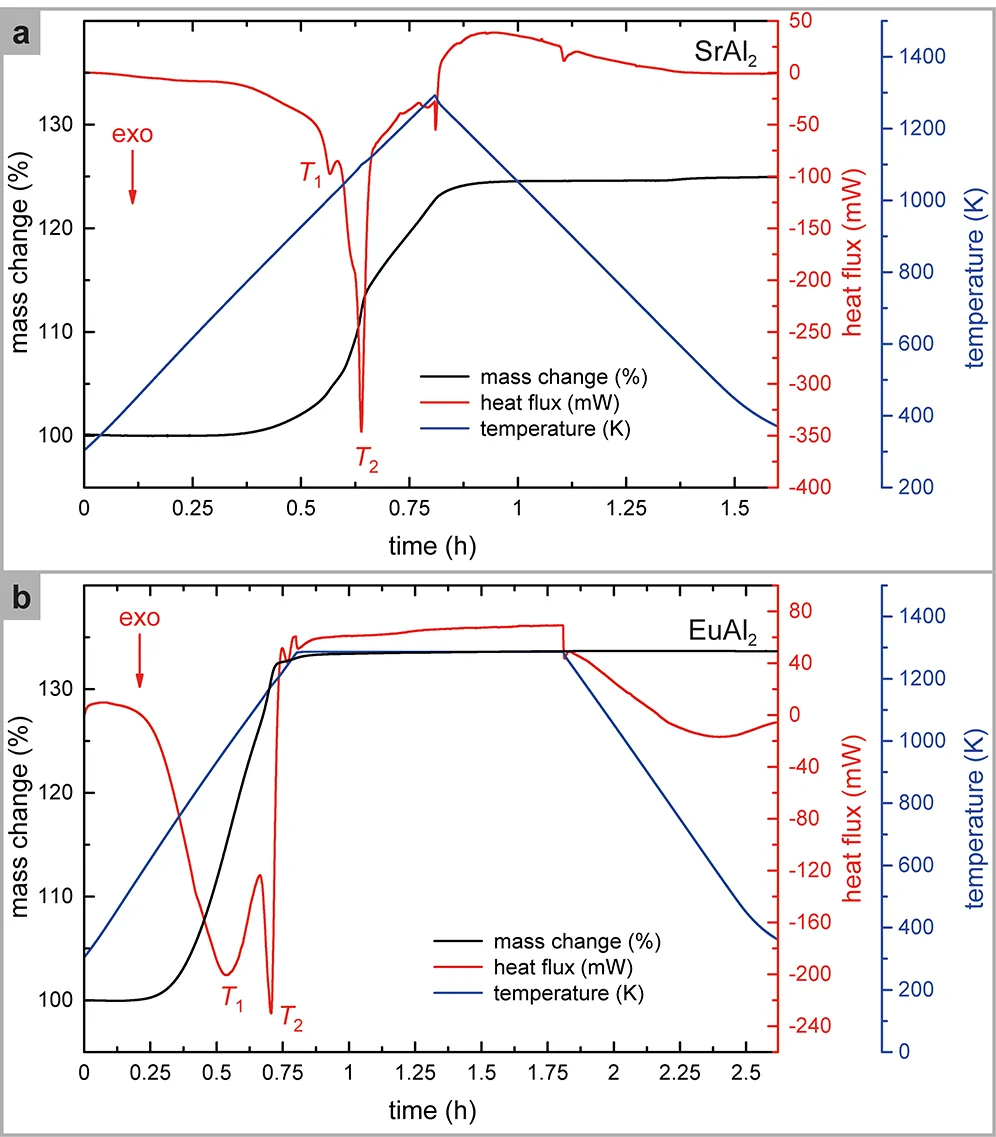
Thermal analysis of (a) SrAl_2_ and (b) EuAl_2_. The mass change is shown in black, the heat flux is shown in red, and the temperature in blue.

For EuAl_2_, a mass increase of 31% is expected when forming Eu^II^Al_2_O_4_, this is at first glance in line with the observed 34%. However, subsequent powder X-ray diffraction (Figure S2b, Table S1) revealed the X-ray pure formation of orthorhombic EuAlO_3_ (GdFeO_3_ type, *Pnma*) instead of the desired monoclinic Eu^II^Al_2_O_4_ (SrAl_2_O_4_ type, *P*2_1_). This is not surprising as europium exhibits two oxidation states (Eu^2+^ and Eu^3+^) in contrast to strontium. With an excess of O_2_, the formation of trivalent Eu is favored leading to the observed Eu^III^AlO_3_. However, this is in clear contrast to the oxidation of EuAl_2_ with elemental sulfur solely leading to EuAl_2_S_4_.^37^ One puzzling aspect of this reaction are the formally missing Al atoms which should form Al_2_O_3_ according to a full oxidation as shown in eq 1.

1 $$4\;{EuAl}_{2}+9\;O_{2}\to 4\;{EuAlO}_{3}+2\;{Al}_{2}O_{3}$$

As the Rietveld refinement of the powder X-ray diffraction data (Figure S2b) indicates no distinct mixing of Al and Eu according to (Eu_0.67_Al_0.33_)­AlO_3_, the suspicion arose that Al either diffuses into the crucible or amorphous aluminum oxide is formed. In the literature, the decomposition of EuAl_2_O_4_ at 1273 K under the O_2_ atmosphere into EuAlO_3_ and Al_2_O_3_ is described, which could also happen here.^39^ To investigate this hypothesis, SEM/EDX, ICP–MS, and ^27^Al NMR spectroscopic measurements were conducted on the X-ray pure sample of EuAlO_3_ (Figure S2b). The EDX results clearly show a ratio of 35(2) at.% Eu to 65(2) at.% Al in line with the precursor composition of EuAl_2_ and ICP–MS measurements of the dissolved samples also gave a reasonable confirmation (Eu/Al = 1:1.86). Oxygen was not determined in the EDX scans due to the limitation of the instrument. Subsequently, ^27^Al solid-state MAS NMR spectra of nominal EuAlO_3_ were collected (Figure 4). Here, three distinct resonances are observed. The signal at 1640 ppm (green) corresponds to elemental Al,^63^ while the resonance at 12 ppm (blue) corresponds to oxidic Al^3+^ species like Al_2_O_3_ since the spectra were referenced to Al^3+^ in aqueous solution (Figure 4a). The elemental Al is observed due to an incomplete oxidation of a EuAl_2_ particle, in line with our observations on the oxidation of CaAl_2_.^33^ A zoomed view of the region near the signal at 12 ppm is shown in Figure 4b. Besides the resonance at 12 ppm also a second, significantly smaller signal at 30 ppm can be observed. When comparing this pattern to the literature, γ-Al_2_O_3_ seems to fit the best, however, a signal at ∼75 ppm is expected which would overlap with the rotational sideband in our spectrum. The signal at 12 ppm can be attributed to [AlO_6_], while the one at 30 ppm can be assigned to [AlO_5_]. Finally, the signal at 201 ppm (red) can be assigned to EuAlO_3_, in line with the resonance reported in literature at 213 ppm.^64^ This leads to the conclusion that the “missing” Al is oxidized forming amorphous alumina. Since the NMR spectroscopic experiments cannot be quantified, no conclusion with respect to the amount of amorphous Al_2_O_3_ can be drawn. Therefore, powder X-ray investigations using elemental Si as an internal standard were conducted (Figure S3). Rietveld refinement clearly shows that these samples contain a high amount of amorphous material (∼65 mass%). This clearly shows that the sample contains amorphous parts, even above the expected amount (∼18 mass%) according to the reaction proposed in eq 1.

**Figure 4:**
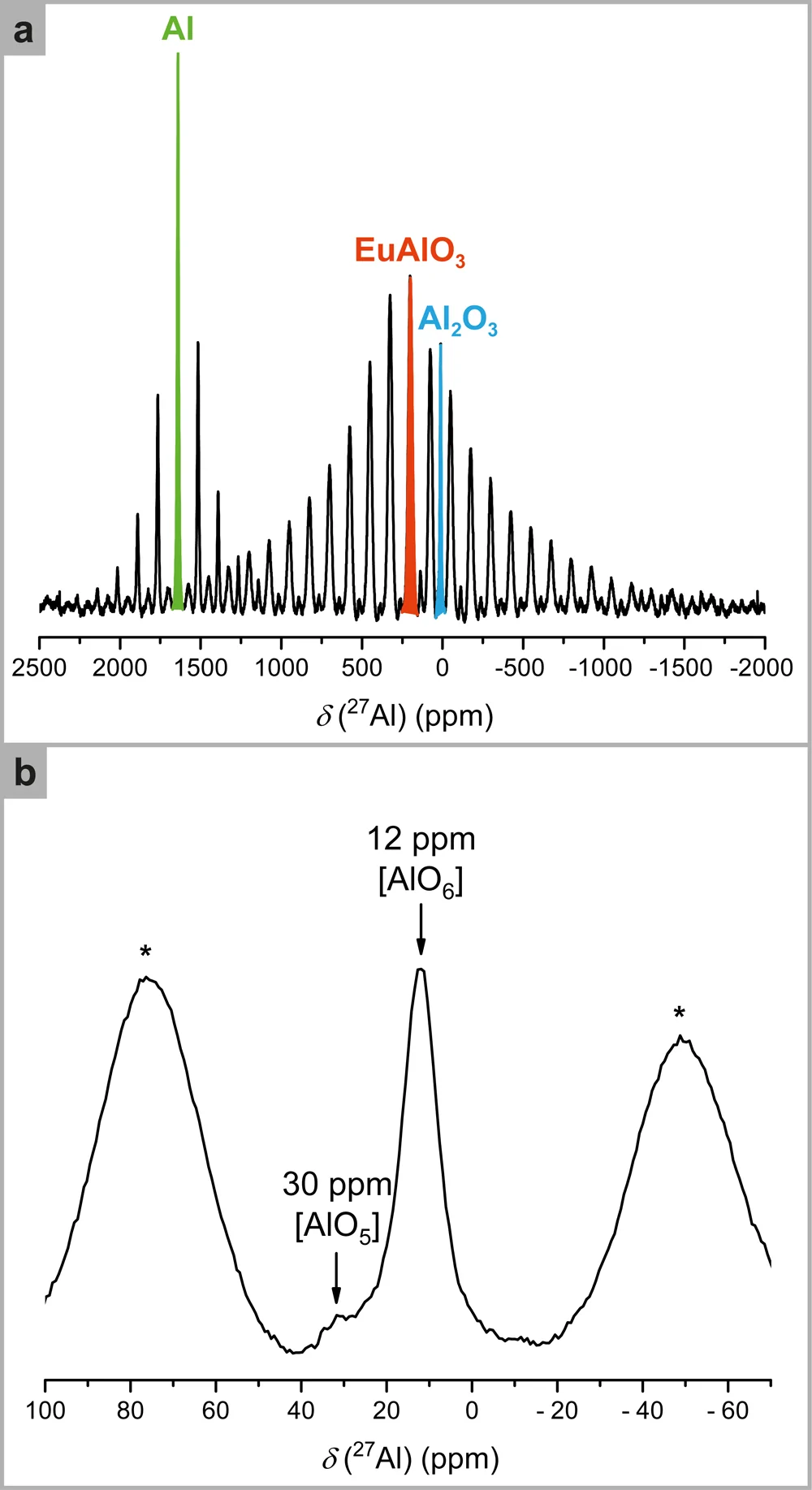
^27^Al solid-state MAS NMR spectra of nominal EuAlO_3_. (a) The visible resonances have been color coded and attributed to EuAlO_3_ (red), elemental Al (green), and Al_2_O_3_ (blue). (b) Zoom on the central transitions of Al_2_O_3_. The intensities do not scale with the respective at.% or mass% fractions in the sample. All other signals are rotational side bands due to the MAS conditions.

Based on the results from the thermal analysis of the EuAl_2_ oxidation, bulk oxidation reactions at different temperatures and for different times were conducted in a flow furnace. As the thermal events happened at *T*_1_ = 980 and *T*_2_ = 1170 K, oxidations were carried out below the first event (at 773 K), between the first and the second event (at 1073 K), and at temperatures above the second event (at 1273 K). When held at 773 K for 5 h, a significant broadening of the reflections of EuAl_2_ is observed associated with a reduction in crystallite size from >200 to 58(1) nm. In addition, the appearance of additional reflections was observed. These could, in part, be matched to EuAl_2_O_4_, underlining the proposed initial formation of the divalent europium aluminate (Figure S4). When oxidized at 1073 K (0 or 5 h), X-ray amorphous samples were obtained (Figure S5a,b), however, dwelling at 1073 K for 48 h led to reflections matching EuAlO_3_ alongside an unidentified phase (Figure S5c). SEM/EDX measurements of the amorphous sample oxidized at 1073 K for 5 h show a ratio of 32(2) at.% Eu to 68(2) at.% Al, again in line with the precursor composition of EuAl_2_.

At 1273 K (0 h), 88(1) mass% EuAlO_3_ are observed along with 5(1) mass% EuAl_2_ and 7(1) mass% Eu_2_O_3_ (bixbyite type, *Ia*3̅). This is consistent with the observations on the oxidation of CaAl_2_ ^33,34^ or ScAl_2_,^35^ where Ca or Sc are oxidized first. In addition, again reflections that could not be assigned to binary intermetallics, binary and ternary oxides are observed (Figure S6), for longer dwell times, the results are consistent with the observations described in the STA experiments above.

In order to synthesize an aluminate containing solely divalent europium, a defined amount of oxygen was used during the oxidation. This was achieved by the thermal decomposition of BaO_2_ (∼773 K) in the presence of EuAl_2_ in evacuated silica ampules. Subsequent heating to 1373 K led to X-ray pure EuAl_2_O_4_ (Figure S2c). Interestingly, the use of stoichiometric amounts of BaO_2_ (4 equiv.) led to an overoxidation; however, the use of 3 eq. of BaO_2_ yielded the X-ray pure sample. This can be explained by adhesive water on the surface of the silica ampules and the alumina crucibles. When the silica ampule is preheated with an open flame under dynamic vacuum, this water can be removed leading to no overoxidation of EuAl_2_ occurs when 4 eq. of BaO_2_ is used.

Finally, attempts to recrystallize the amorphous γ-Al_2_O_3_ were made. Here, the sample oxidized at 1273 K for 5 h containing EuAlO_3_ and amorphous γ-Al_2_O_3_ was pressed into a pellet (Ø 6 mm) and sealed in an evacuated silica tube and annealed for 48 h. Surprisingly, the initially white sample turned green. The powder X-ray diffraction pattern revealed the formation of EuAl_2_O_4_ (54(1) mass%) alongside remaining EuAlO_3_ (46(1) mass%). This indicates that the following reaction takes place

2 $$4\;{EuAlO}_{3}+2\;{Al}_{2}O_{3}⇌4\;{EuAl}_{2}O_{4}+O_{2}$$

The formation of oxygen is favored due to the evacuated ampule. Subsequently, the sample was sealed in an evacuated silica tube alongside Ti sponge. This led to the formation of almost X-ray pure EuAl_2_O_4_ (97(1) mass%; Figure S7) underlining the reaction given in eq 2. This is also in line with the energies of formation extracted from the OQMD.^65^ Here, EuAl_2_O_4_ has a higher formation energy of −3.405 eV atom^–1^ versus −3.106 eV atom^–1^ for EuAlO_3_, rendering the first compound more stable.

### 3.3 Photoluminescence

While EuAl_2_ shows metallic luster as a melting bead, it is a blackish powder when ground. The oxidized samples that were identified as EuAlO_3_ are colorless under white light, EuAl_2_O_4_ appears dark green (Figure 5). UV–vis measurements were performed to investigate the optical band gap (Figure S8). While EuAl_2_O_4_ shows a band gap of 2.67 eV in line with the observed green color and the literature,^39^ EuAlO_3_ exhibits an optical band gap of 3.94 eV, in line with the white appearance. Under UV light with λ = 365 nm, EuAlO_3_ shows a red and EuAl_2_O_4_ a bright green luminescence in line with the literature and the expected trivalent and divalent oxidation states, respectively (Figure 5). Photoluminescence spectra were recorded on both EuAlO_3_ and EuAl_2_O_4_. In line with the expectation, Eu^III^AlO_3_ containing trivalent europium emits at 613 nm (excitation wavelength 395 nm), in line with the literature (∼615 nm),^66^ with rather narrow lines (Figure S9a) originating from the f→f (^5^D_0_→^7^F_2_) transitions. For Eu^II^Al_2_O_4_, a broad emission with a maximum at 520 nm (excitation wavelength 375 nm) was observed (Figure S9b). Also, this is in line with reports in the literature both in the pure Eu-compound^67,68^ or Eu-substituted SrAl_2_O_4_.^69^ In the excitation spectrum, a bump at 395 nm is visible, indicating traces of trivalent europium not visible in the powder X-ray diffraction (*vide supra*) or magnetism (*vide infra*). The green color of the emission of Eu^II^Al_2_O_4_ was attributed to the special surrounding of the Eu atoms. The crystal field will force the d-orbitals to orient in a specific direction, leading to lowering of its energy which in turn leads to a shift of the Eu^2+^ emission to longer wavelengths.^69^

**Figure 5:**
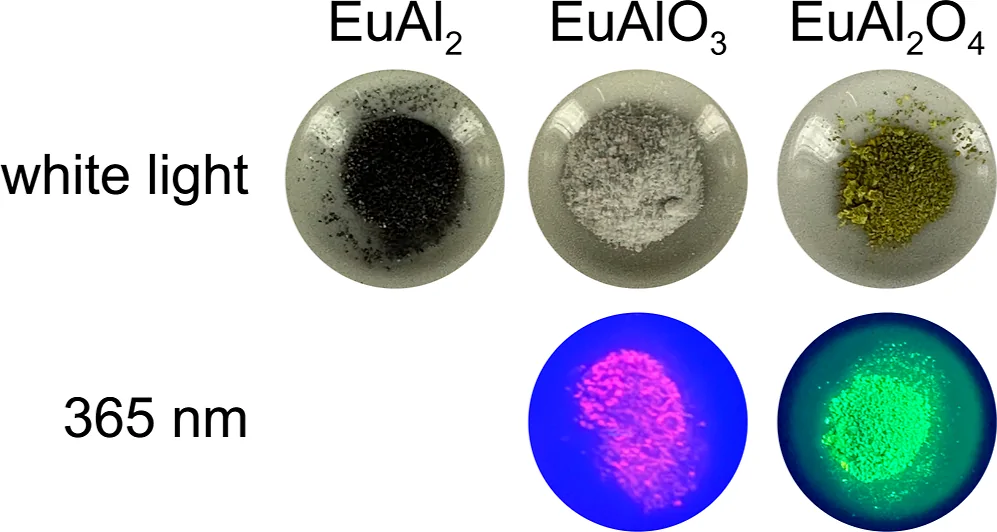
Photographs of EuAl_2_ (blackish), EuAlO_3_ (white), and EuAl_2_O_4_ (greenish) under white light and UV light with 365 nm. The red luminescence of trivalent Eu in Eu^III^AlO_3_ and green luminescence of divalent Eu in Eu^II^Al_2_O_4_ is clearly visible.

### 3.4 Magnetic Properties

The different oxidation states of the Eu atoms in Eu^III^AlO_3_ and Eu^II^Al_2_O_4_ were furthermore investigated by magnetic susceptibility measurements. Eu^2+^ is isoelectronic to Gd^3+^ (4f^7^) and therefore exhibits strong paramagnetism and a theoretical magnetic moment of μ_theo_ = 7.94 μ_B_.^8,9^ Eu^3+^ ([Xe] 4f^6^, *S* = 3, *L* = 3) in contrast is a so-called van Vleck ion with a theoretical magnetic moment of μ_theo_ = 0 μ_B_ due to spin–orbit coupling. The lowest state, however, exhibits a multiplet splitting (^7^F_*J*_) with the ground state being ^7^F_0_. The calculated moment of 0 μ_B_ is only true for this ground state. For higher temperatures, the different excited states of the multiplet must be taken into account. The paramagnetic susceptibility therefore has to be expressed by the (modified) van Vleck theory^10^

$$\chi_{para}\left(free\;{Eu}^{3+}\right)=\frac{N\mu_{B}^{2}}{Z}\left(\frac{A}{3\lambda }\right)+n\left(\frac{C}{T}\right)+\chi_{0}$$

The expressions of *A* and *Z* contain the contributions of the respective possible ground states ^7^F_0_ to ^7^F_6_ and their respective thermal occupation probability, while λ describes the coupling constant of the ground state (^7^F_0_) and the first excited state (^7^F_1_).^11^ The second term describes the contribution of paramagnetic impurities, e.g., from traces of Eu^2+^ ions while χ_0_ describes the temperature independent diamagnetic contribution from Al_2_O_3_. Here, the diamagnetic increments^70^ were used. Figure 6a shows the magnetic susceptibility of EuAlO_3_. The plateau is the aforementioned feature of the van Vleck paramagnetism. At low temperatures, an increase is visible, originating from traces of Curie paramagnetic impurities. The red line indicates the fit leading to λ = 592(1) K with a contribution of 0.005(1)% Eu^2+^ and χ_0_ = 5.4 × 10^–4^ emu mol^–1^. For single crystals of EuAlO_3_, a triple degenerate ^7^F_1_ state was observed with the levels 281, 359, and 479 cm^–1^.^71^ These correspond to 404, 516, and 689 K. Due to the polycrystalline nature of our sample, the observed value of λ is in between the values reported in the literature. In comparison, EuF_3_ has a value of λ = 490 K,^11^ EuBO_3_ of λ = 471 K,^11^ and the pyrochlore Eu_2_Ta_2_O_6_N a value of λ = 390 K.^72^ Intermetallic Eu_2_Al_9_Ir_3_ (λ = 380 K^73^) and metallic Eu have lower values (λ = 285 K^3^).

**Figure 6:**
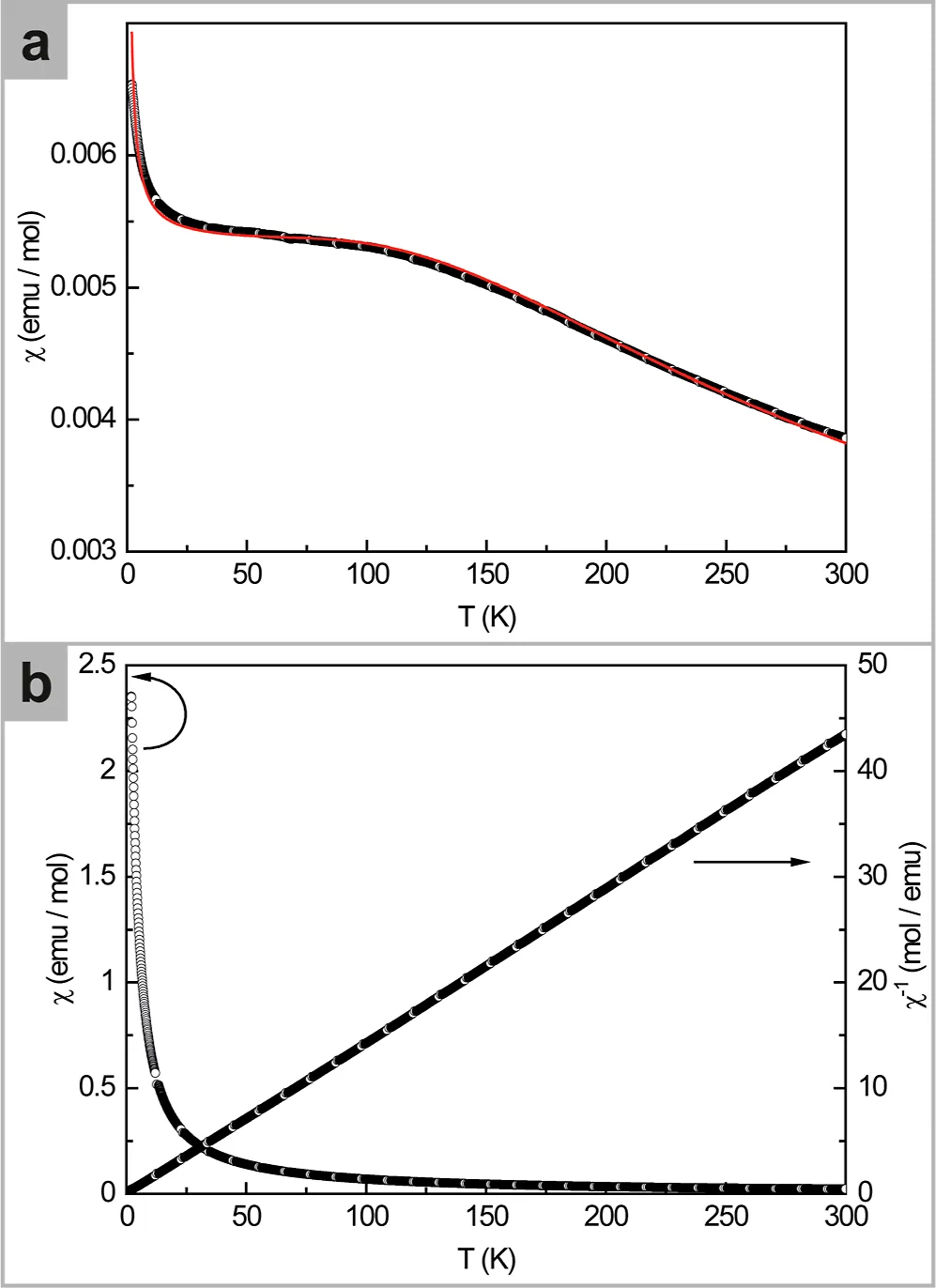
Temperature dependence of the magnetic susceptibility of (a) EuAlO_3_ and (b) EuAl_2_O_4_. The red trace in (a) shows the van Vleck fit, for details see the text.

EuAl_2_O_4_ is, as mentioned above, a Curie paramagnet. The magnetic susceptibility χ and its inverse χ^–1^ are shown in Figure 6b. The inverse susceptibility is linear, and no anomalies can be seen down to 2 K, in line with previous investigations on EuAl_2_O_4_.^74^ The effective magnetic moment was determined to 7.46(1) μ_B_ which is below the theoretical value of μ_theo_ = 7.94 μ_B_, however, still proving that the Eu atoms are predominantly in the divalent state. The deviation can be explained by trace impurities not visible in the powder X-ray diffractograms. The Weiss temperature is 1.0(1) K, indicating very weak interactions, in line with the absence of magnetic ordering and the report in literature.^74^

### 3.5 ^151^Eu Mössbauer Spectroscopic Investigations

To prove the oxidation states of the Eu atoms in EuAlO_3_ and EuAl_2_O_4_ and get additional information about the purity of the samples, ^151^Eu Mössbauer spectra were recorded at 78 K. The obtained fitting parameters are listed in Table S3. The spectrum of EuAlO_3_ could be fitted with one signal (Figure 7a), for EuAl_2_O_4_, two signals (Figure 7b) were necessary. This is in line with the respective crystal structures (*vide supra*). The signal of EuAlO_3_ exhibits an isomer shift of δ = +0.74(2) mm s^–1^, in line with Eu in the oxidation state +3 and an oxidic environment.^15,75,76^ Additionally, no hyperfine splitting is observed, underlining the absence of magnetic ordering. The two signals of EuAl_2_O_4_ show isomer shifts of δ = −11.28(7) mm s^–1^ and −14.07(3) mm s^–1^. They can be attributed to Eu^2+^,^15,75,76^ again without any signs of magnetic ordering. The observed shifts are furthermore in the same range as the ones reported in the literature.^39^ Because of the noncubic site symmetry of the europium atoms, the signals of all spectra show quadrupole splitting (Table S3). The experimental line widths are between 2.9 and 3.3 mm s^–1^, which is a typical range.^21,77–82^ This leads to the conclusion that EuAlO_3_ exhibits tri- and EuAl_2_O_4_ divalent Eu, respectively, and no impurities based on the sensitivity of Mössbauer spectroscopic investigations are present.

**Figure 7:**
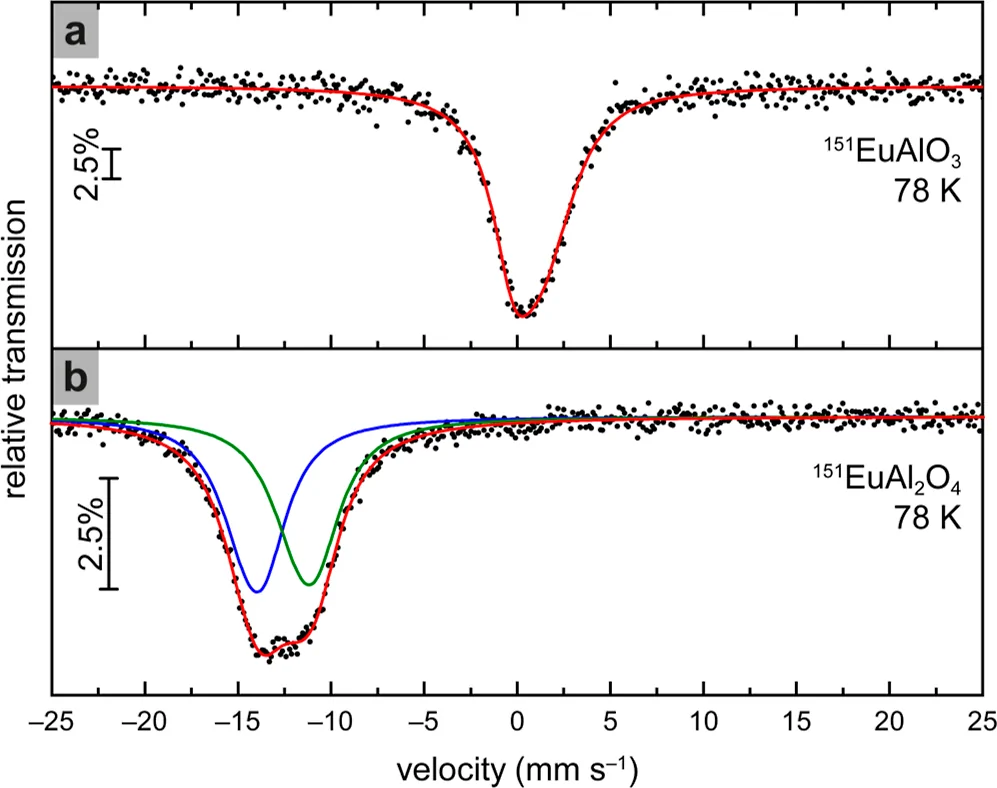
Experimental (data points) and simulated (continuous lines) ^151^Eu Mössbauer spectra of (a) EuAlO_3_ and (b) EuAl_2_O_4_, both measured at 78 K. The red line corresponds to the (a) fit and (b) sum of the different signals used for fitting; the blue and green line correspond to the two crystallographically independent Eu sites.

## 4 Conclusion

In the presented study, SrAl_2_ and EuAl_2_, two intermetallic compounds, were used as precursors for the synthesis of oxides. SrAl_2_ behaves similar to CaAl_2_ and forms a mixture of different compounds based on the synthetic conditions. Using EuAl_2_, however, gives the possibility to adjust the oxidation state of the Eu atoms after oxidation and therefore also the obtained product. As demonstrated, excess O_2_ leads, depending on time and temperature, to the formation of X-ray pure Eu^III^AlO_3_. However, based on the composition of the precursor, a side product must be formed. ^27^Al solid-state NMR spectroscopic investigations exhibit resonances that can be assigned to γ-Al_2_O_3_ which has to be amorphous. Annealing of these samples under vacuum led to the formation of Eu^II^Al_2_O_4_, indicating that Eu^III^AlO_3_ in the presence of Al or Al_2_O_3_ leads to the formation of the divalent Eu-species. Direct synthesis of Eu^II^Al_2_O_4_ is also possible using defined amounts of O_2_. Spectroscopic, magnetic, ^151^Eu Mössbauer spectroscopy, and optical investigations consistently confirm the distinct oxidation states and properties of both compounds.

The results clearly indicate that the expected differences in the oxidation behavior of SrAl_2_ and EuAl_2_, based on the possible oxidation states, exist. With EuAl_2_O_4_, a luminescent material with a green emission has been prepared paving the way for the synthesis of other luminescent materials. The use of solid solutions, e.g. Sr_1–*x*_Eu_*x*_Al_2_ might even enable the synthesis of substituted host structures.

## Supplementary Material



## Data Availability

The data that supports the findings of this study are available from the corresponding author upon reasonable request.

## Notes

Diffraction patterns, diffuse reflectance spectra, emission and excitation spectra, crystallographic data, SEM/EDX results, ^151^Eu Möβbauer spectroscopic data, and Chardi values (PDF)
