# Supplementary material for: Oxidizing EuAl2: A Precursor for the Synthesis of Di- and Trivalent Europium Oxides
Source: Inorg Chem. 2026 Jul 31;65(32):18938–47. doi: 10.1021/acs.inorgchem.6c02830 (PMC13488440; doi:10.1021/acs.inorgchem.6c02830)
Supplement: Supplementary file 1 [file ic6c02830_si_001.pdf]

SUPPORTING INFORMATION

Cedric Kloos,[a,#] Elias C. J. Gießelmann,[a,#] Aaron Haben,[b,c] Lena Ruck,[a] Jasper A. Baldauf,[d] Joshua Wiethölter,[d] Rainer Pöttgen,[d] Guido Kickelbick,[a] Oliver Janka[a,*]

Oxidizing $EuAl_2$: A Precursor for the Synthesis of Di- and Trivalent Europium Oxides

[a] Saarland University, Solid State Inorganic Chemistry, Campus C4 1, 66123 Saarbrücken, Germany

[b] Saarland University, WASTe - Elemental Analysis Group, Campus C4 1, 66123 Saarbrücken, Germany

[c] Saarland University, Core Facility ELMAS-CF, Campus C4 1, 66123 Saarbrücken, Germany

[d] Universität Münster, Institut für Anorganische und Analytische Chemie, Corrensstraße 30, 48149 Münster, Germany

* Corresponding author: Oliver Janka, Saarland University, Solid State Inorganic Chemistry, Campus C4 1, 66123 Saarbrücken, Germany; E-mail: oliver.janka@uni-saarland.de

[#] Both authors contributed equally.

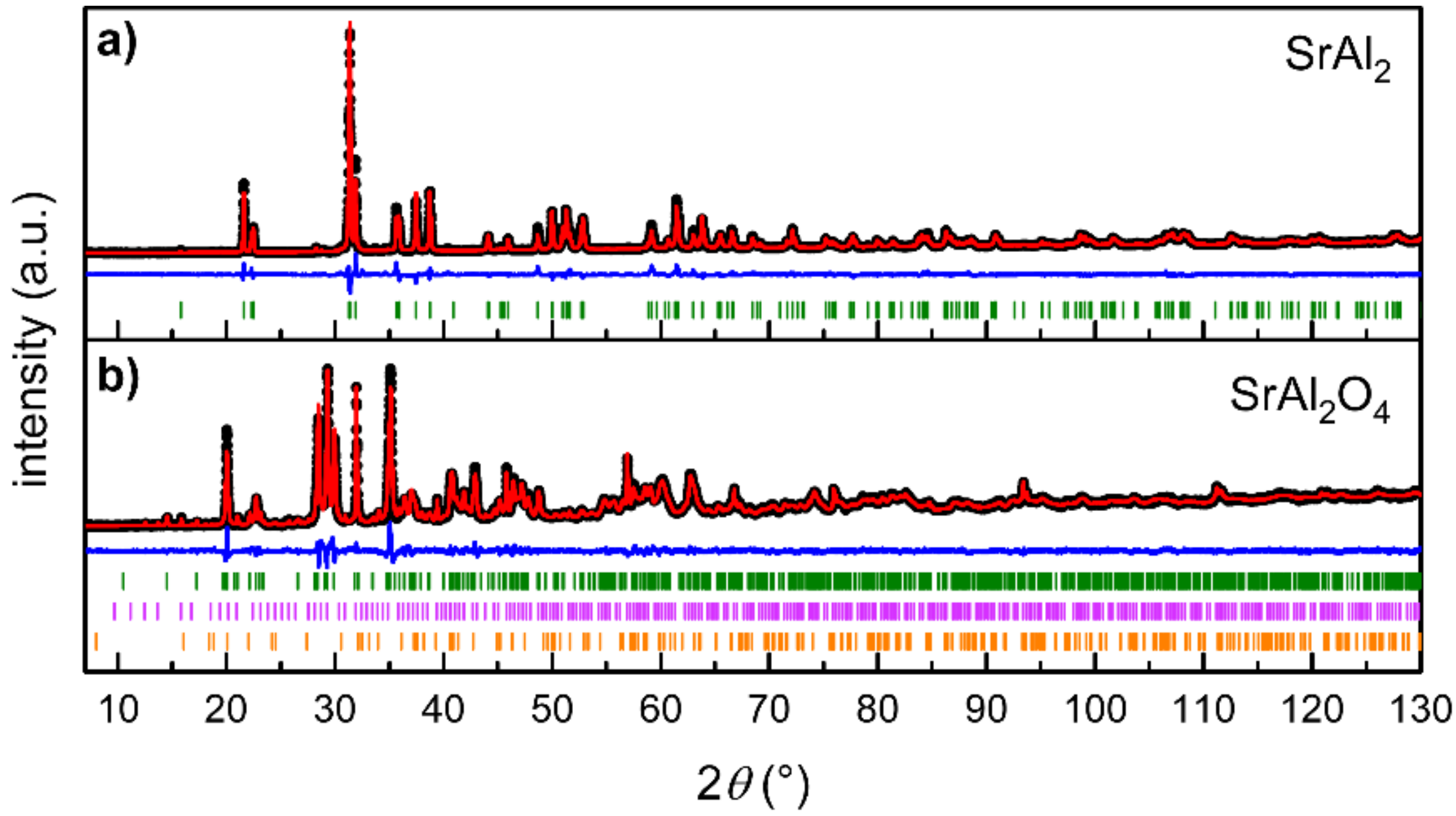

Figure S1. Rietveld refinements of different powder X-ray diffraction patterns. a) precursor $SrAl_2$ (KHg_2 type, *Imma*) b) oxidation product $SrAl_2O_4$ (own type, $P2_1$) alongside side products. Collected data is shown as black dots, the refinement as red, the difference as blue line. The colored ticks indicate the Bragg positions of the respective phases. In b) the colors stand for $SrAl_2O_4$ (green), $Sr_3Al_2O_6$ (pink) and $SrAl_{12}O_{19}$ (orange).

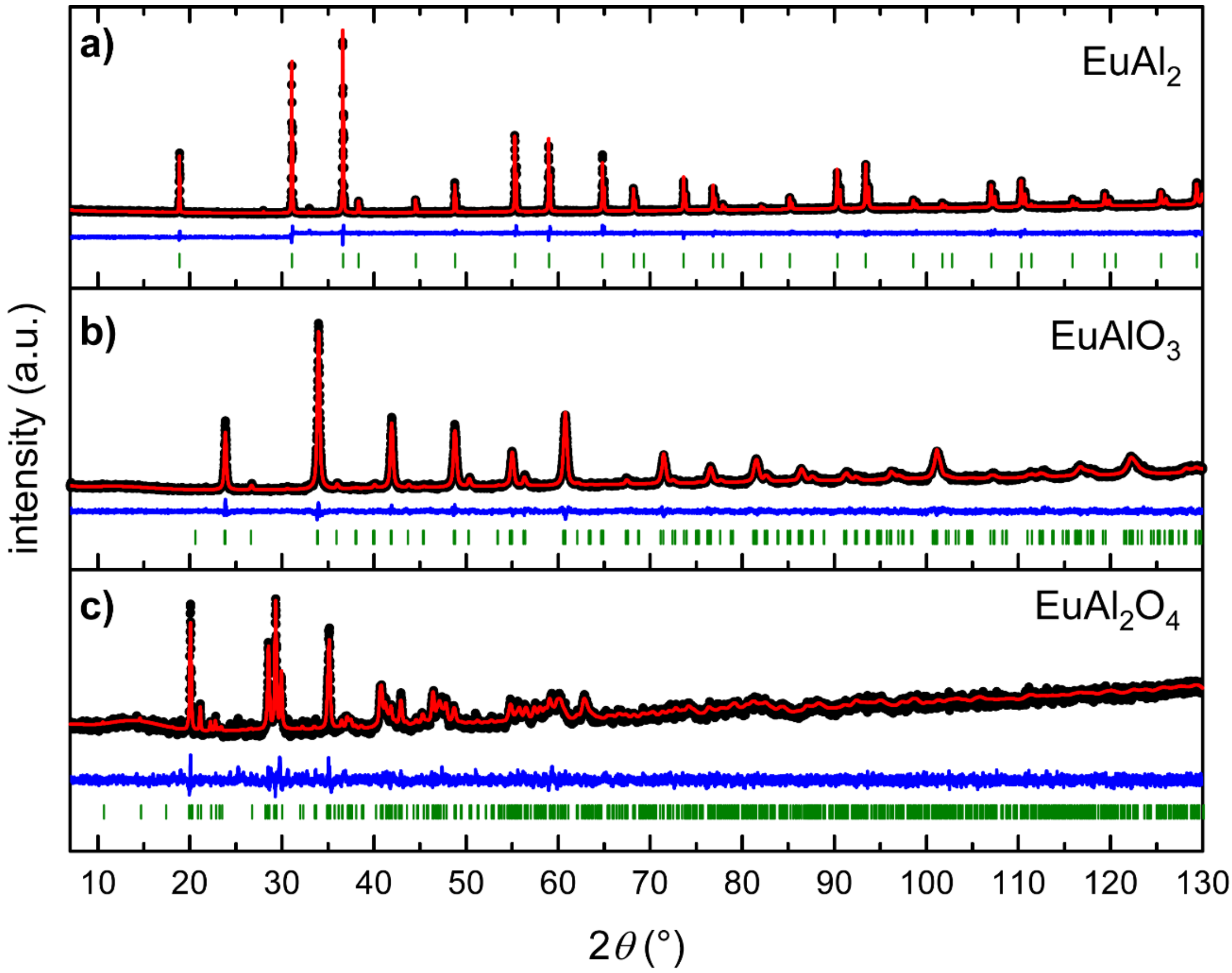

Figure S2. Rietveld refinements of different powder X-ray diffraction patterns. a) precursor $EuAl_2$ ($MgCu_2$ type, $Fd\bar{3}m$), b) oxidation product $Eu^{III}AlO_3$ ($GdFeO_3$ type, *Pnma*) and c) oxidation product $Eu^{II}Al_2O_4$ ($SrAl_2O_4$ type, $P2_1$). Collected data is shown as black dots, the refinement as red, the difference as blue line. Green ticks indicate the Bragg positions of the respective phases.

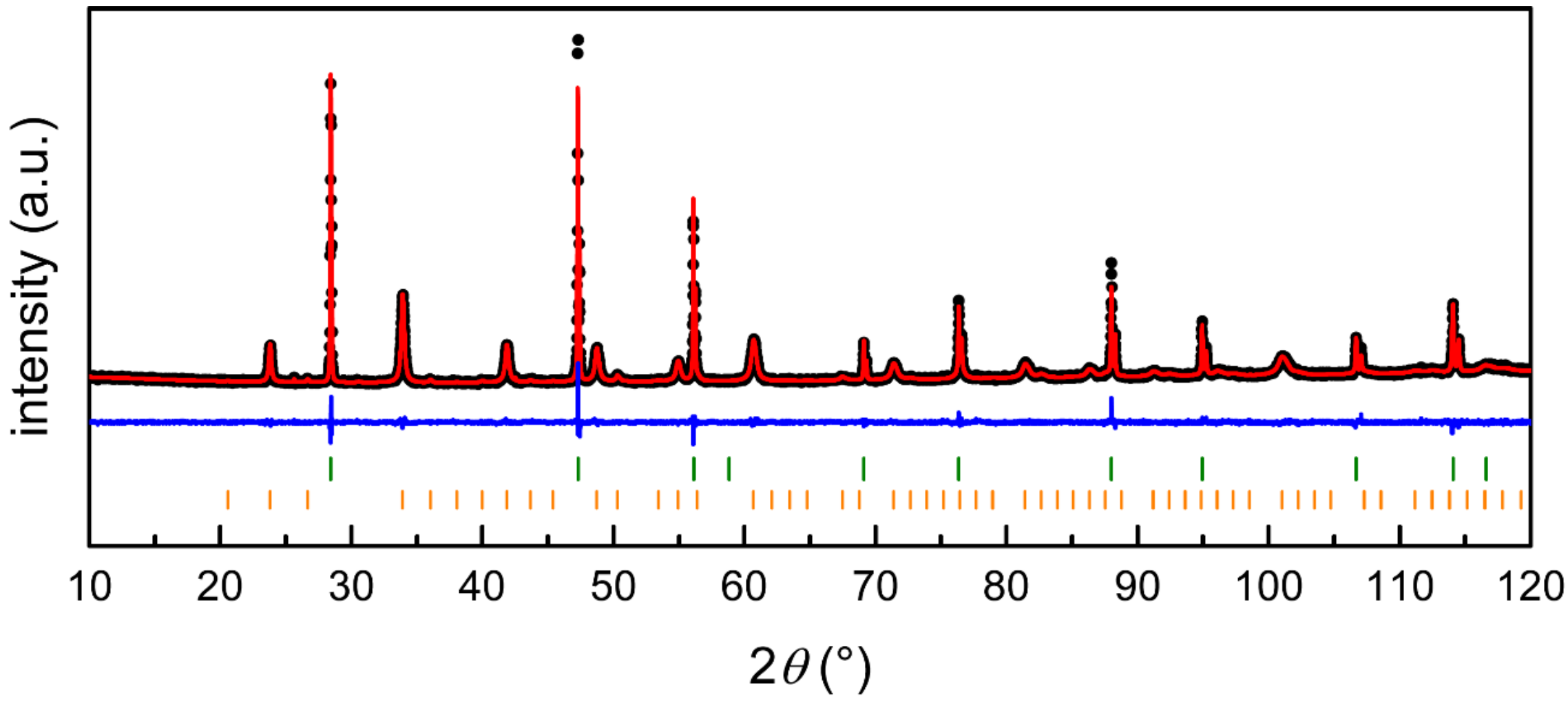

Figure S3. Rietveld refinements of a powder X-ray diffraction pattern of nominal $Eu^{III}AlO_3$ mixed with elemental silicon to determine the amorphous fraction. Collected data is shown as black dots, the refinement as red, the difference as blue line. Green ticks indicate the Bragg positions of $Eu^{III}AlO_3$, orange ticks of elemental Si.

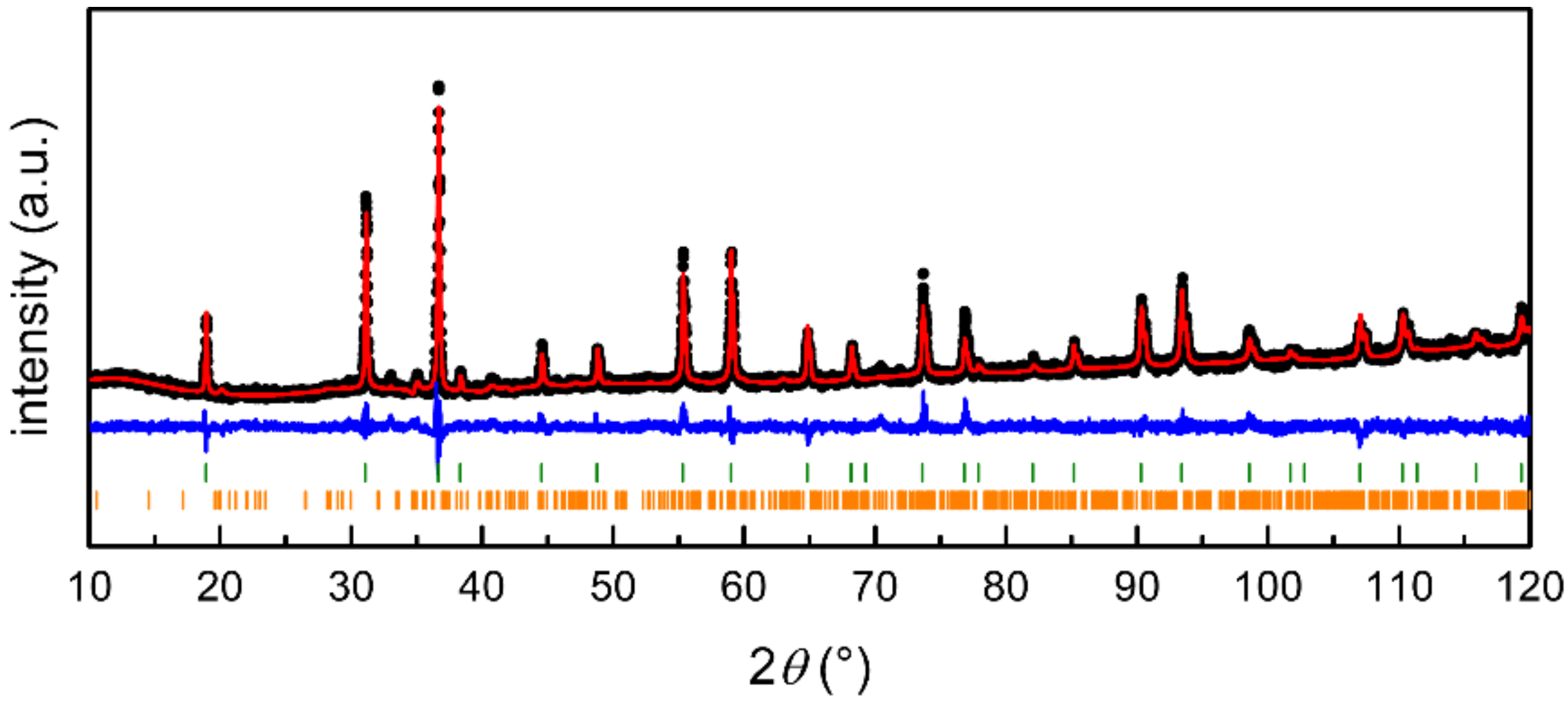

Figure S4. Rietveld refinements of the powder X-ray diffraction pattern of a sample annealed at 773 K for 5 h. Collected data is shown as black dots, the refinement as red, the difference as blue line. Green ticks indicate the Bragg positions of $EuAl_2$, orange ticks of $Eu^{II}Al_2O_4$.

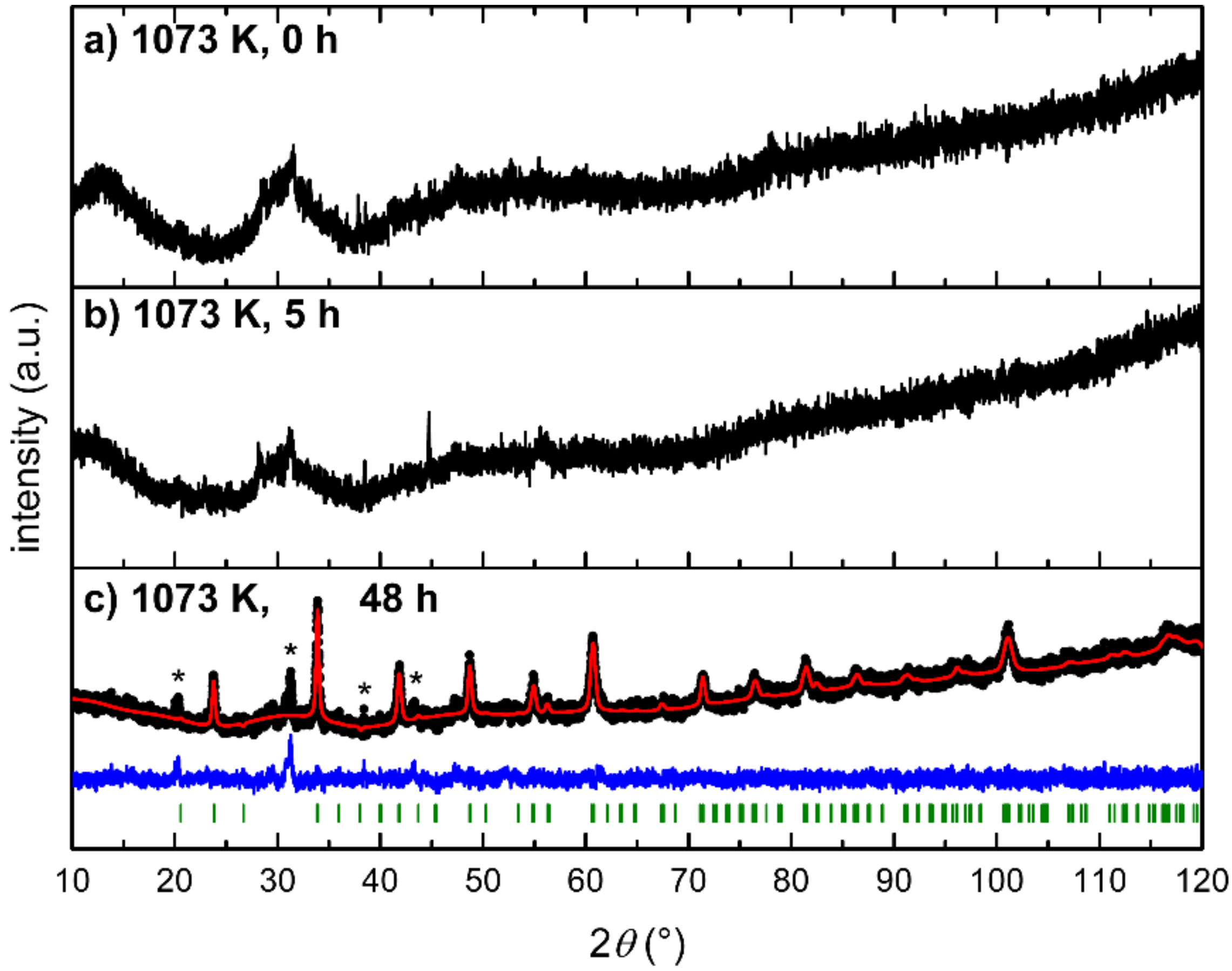

Figure S5. Rietveld refinements of the powder X-ray diffraction patterns of a sample annealed at 1073 K for a) 0 h, b) 5 h and c) 48 h. Collected data is shown as black dots, the refinement as red, the difference as blue line. Green ticks indicate the Bragg positions of $Eu^{II}Al_2O_4$, and asterisk mark an unidentified phase.

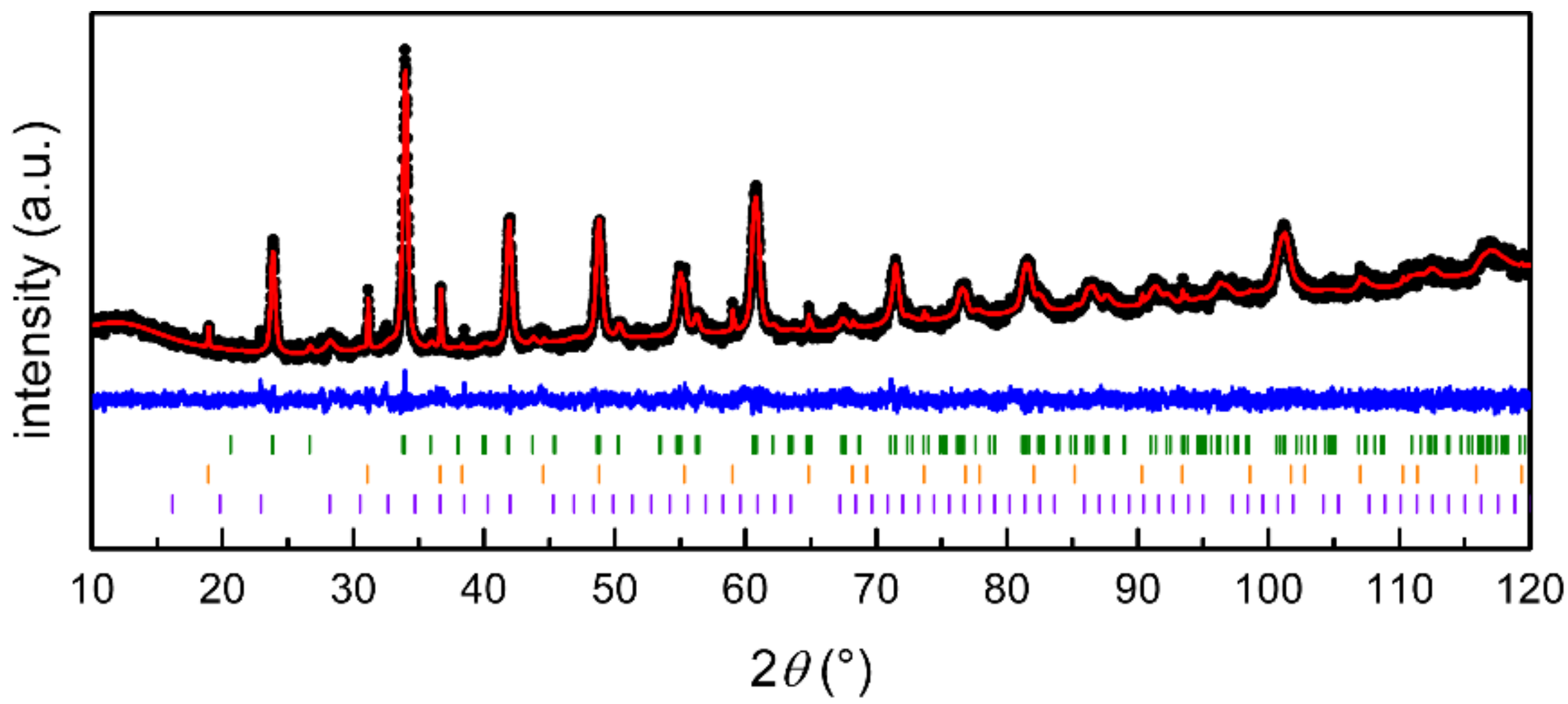

Figure S6. Rietveld refinements of the powder X-ray diffraction pattern of a sample annealed at 1273 K for 0 h. Collected data is shown as black dots, the refinement as red, the difference as blue line. Green ticks indicate the Bragg positions of $Eu^{III}AlO_3$, orange ticks of $EuAl_2$ and purple ticks of Eu_2O_3.

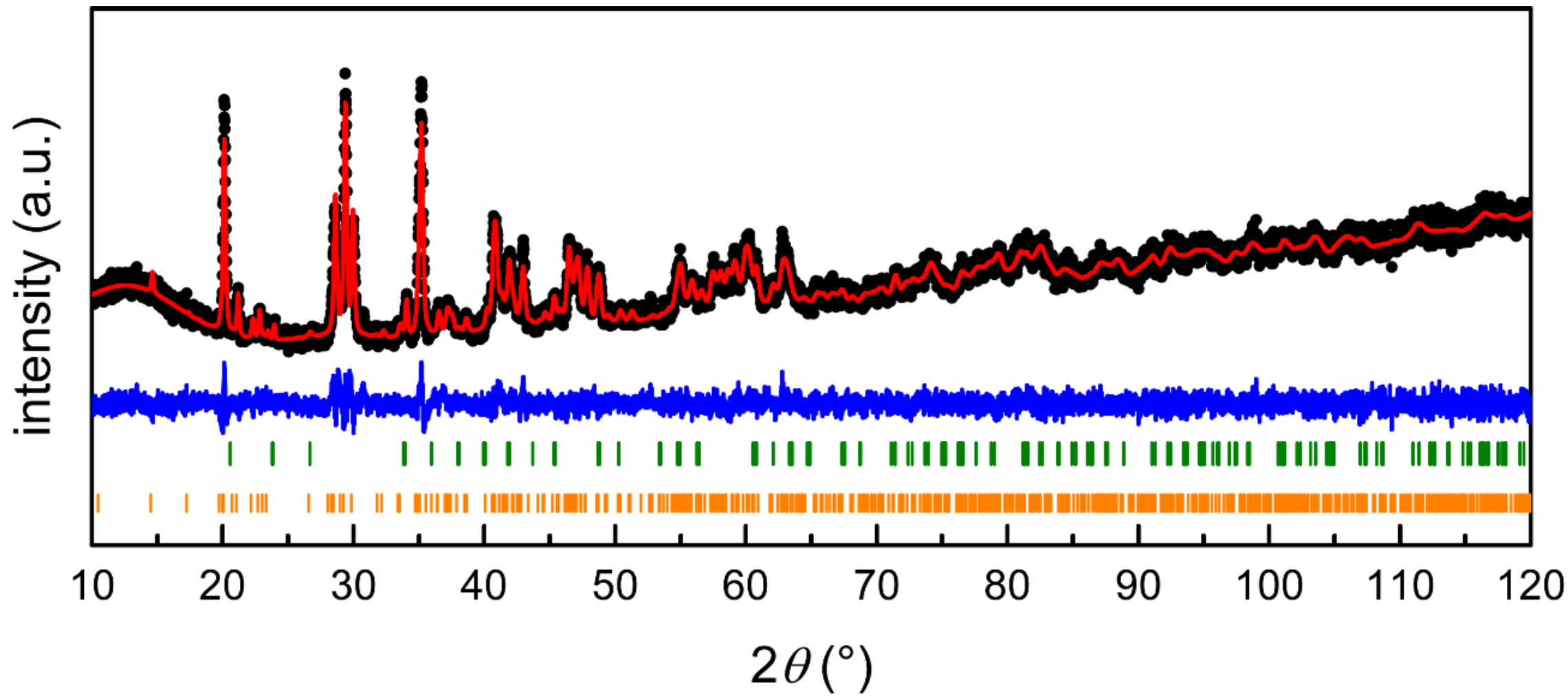

Figure S7. Rietveld refinements of the powder X-ray diffraction pattern of a sample annealed at 1273 K for 64 h under vacuum. Collected data is shown as black dots, the refinement as red, the difference as blue line. Green ticks indicate the Bragg positions of $Eu^{III}AlO_3$, orange ticks of $Eu^{II}Al_2O_4$.

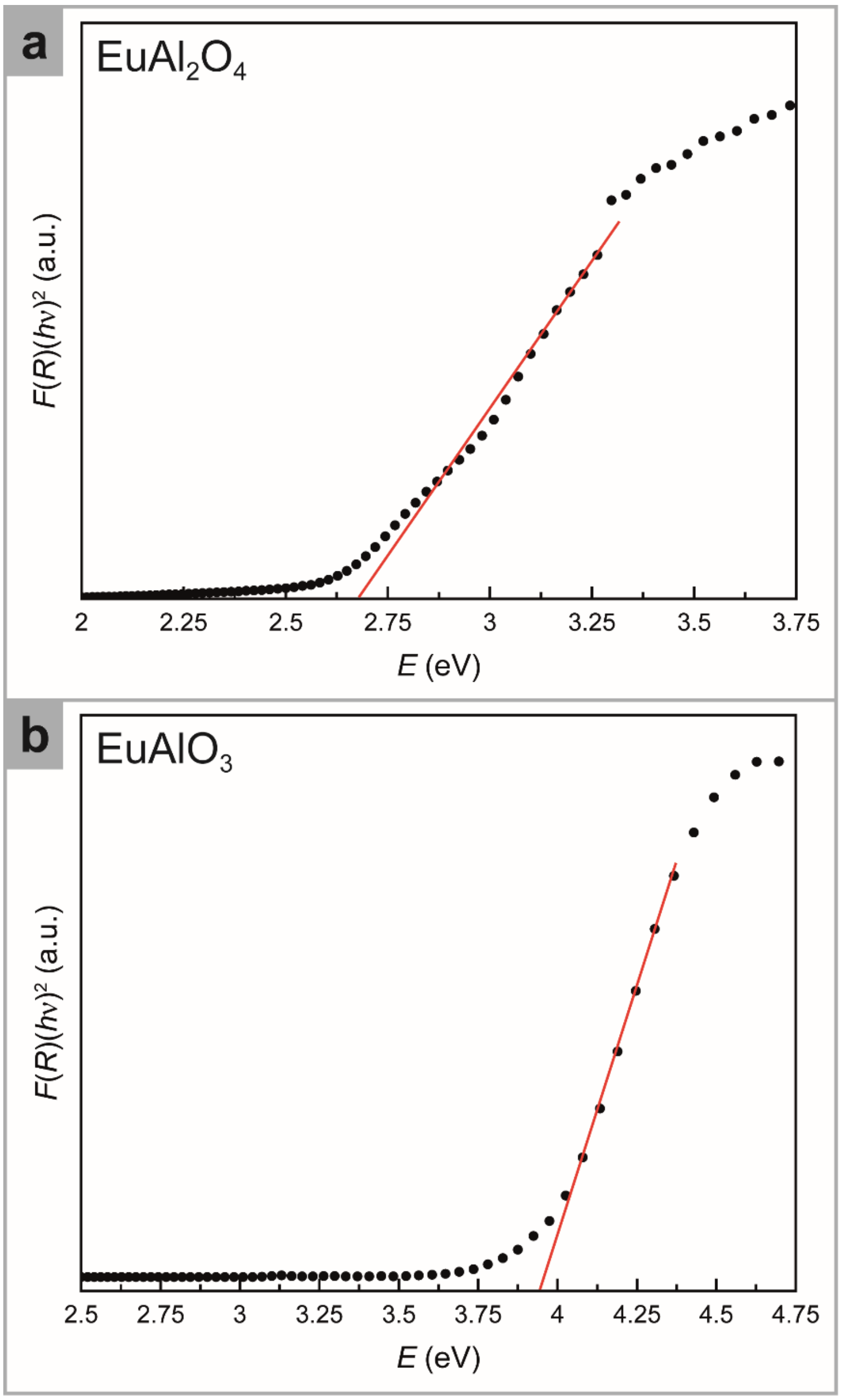

Figure S8. Diffuse reflectance spectra of (a) $Eu^{II}Al_2O_4$ and (b) $Eu^{III}AlO_3$ presented as Tauc plots. The linear parts of the plots were extrapolated to the *x* axes for band gap determination.

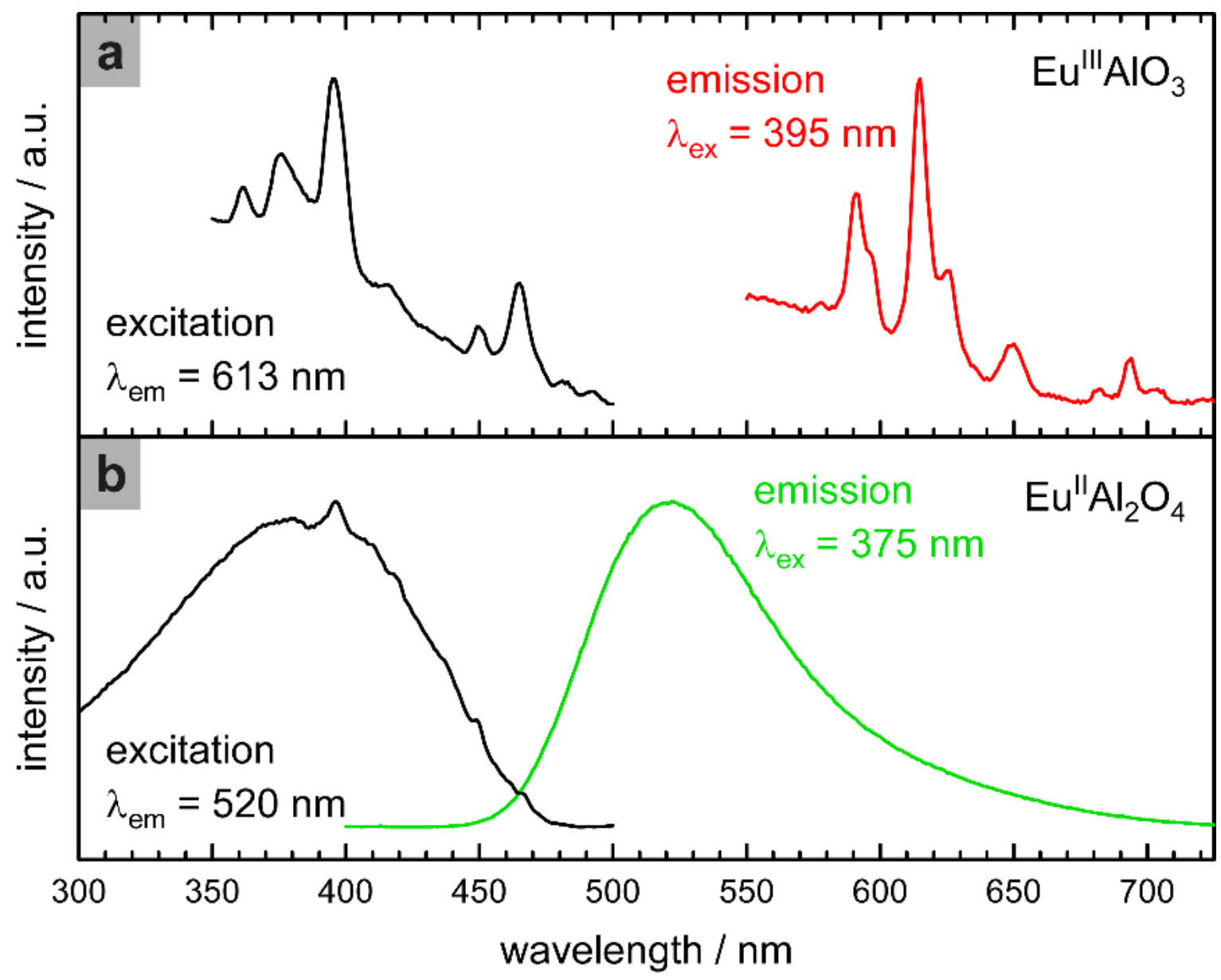

Figure S9. Emission and excitation spectra of a) $Eu^{III}AlO_3$ and b) $Eu^{II}Al_2O_4$. The excitation and emission wavelength are given.

Table S1. Lattice parameters, refined from powder X-ray diffraction, of $SrAl_2$ (KHg_2 type, $Z = 4$), $SrAl_2O_4$ (own type, $Z = 4$), $EuAl_2$ ($MgCu_2$ type, $Z = 8$), $Eu^{II}Al_2O_4$ ($SrAl_2O_4$ type, $Z = 4$) and $EuAlO_3$ ($GdFeO_3$ type, $Z = 4$) alongside literature data.

Compound	space group	a / pm	b / pm	c / pm	β / °	V / nm^3	ref.
$SrAl_2$	*Imma*	484	792	799	90	0.3063	[1]
$SrAl_2$	*Imma*	479.62	789.30	795.51	90	0.3012	[2]
$SrAl_2$	*Imma*	479.56	789.52	795.92	90	0.3014	*
$SrAl_2O_4$	$P2_1$	516.3	881.6	844.7	93.42	0.3838	[3]
$SrAl_2O_4$	$P2_1$	514.72	883.95	844.49	93.297	0.386	*
$EuAl_2$	$Fd\bar{3}m$	812.5	*a*	*a*	90	0.5364	[4]
$EuAl_2$	$Fd\bar{3}m$	812.8	*a*	*a*	90	0.5370	[5]
$EuAl_2$	$Fd\bar{3}m$	812.88	*a*	*a*	90	0.5371	*
$Eu^{II}Al_2O_4$	$P2_1$	515.64	882.39	844.48	93.185	0.3836	[6]
$Eu^{II}Al_2O_4$	$P2_1$	514.61	884.41	844.79	93.077	0.3838	*
$Eu^{III}AlO_3$	*Pnma*	529.2	745.8	527.1	90	0.2080	[7]
$Eu^{III}AlO_3$	*Pnma*	529.72	745.50	527.32	90	0.2082	*

* this work.

Table S2. Chemical compositions determined by SEM/EDX for $EuAl_2$ and the oxidation products.

Reaction	nominal composition	theoretical composition (at%) Eu	Al	experimental composition (at%) Eu	Al
$EuAl_2 + 2\ O_2$	$Eu^{II}Al_2O_4$	33.3	66.7	34(2)	66(2)
$EuAl_2$ + excess O_2	$EuAlO_3$	33.3	66.7	35(2)	65(2)

Table S3. Fitting parameters of the ^{151}Eu Mössbauer spectra of $EuAl_2$ ($MgCu_2$ type, space group $Fd\bar{3}m$, $Z = 8$), $Eu^{II}Al_2O_4$ ($SrAl_2O_4$ type, space group $P2_1$, $Z = 4$) and $EuAlO_3$ ($GdFeO_3$ type, space group *Pnma*, $Z = 4$) measured at 78 K. δ: isomer shift; Γ: experimental line width; ΔE_Q: quadrupole splitting.

Compound	T (K)	site	δ (mm s^{-1})	Γ (mm s^{-1})	ΔE_Q (mm s^{-1})	B_{hf} (T)	ref.
$Eu^{II}Al_2$	78		–8.38(2)	2.96(7)	–	–	[5]
	6		–8.44(3)	2.55(3)	–	25.9(1)	[5]
	4.2		–9.7	–	–	27.8(8)	[8, 9]
$Eu^{II}Al_2O_4$	RT	Eu1	–13.40(10)	2.84(12)	–15.6(4)^b	–	[6]
		Eu2	–12.83(13)	2.0(5)	16.4(8)^b	–	[6]
	78	Eu1 (50%)^a	–14.07(3)	3.2(2)	2.7(6)	–	*
		Eu2 (50%)^a	–11.28(7)	3.3(2)	2.8(6)	–	*
$Eu^{III}AlO_3$	78		+0.74(2)	2.90(9)	–4.6(2)	–	*

* this work; ^{a} fixed ratio according to crystal structure, ^{b} these values are given as $eV_{zz}Q_g$.

Table S4. Chardi values[10, 11] for the individual atoms in $Eu^{II}Al_2O_4$ and $Eu^{III}AlO_3$.

$Eu^{II}Al_2O_4$	Eu1	Eu2	Al1	Al2	Al3	Al4		
	+1.94	+2.10	+3.21	+3.10	+2.64	+3.01		
$Eu^{II}Al_2O_4$	O1	O2	O3	O4	O5	O6	O7	O8
	–2.28	–1.86	–1.98	–2.17	–1.83	–2.13	–2.05	–1.71
$Eu^{III}AlO_3$	Eu	Al	O1	O2				
	+3.00	+3.00	–1.96	–2.07				

References

[1] G. Nagorsen, H. Posch, H. Schäfer, A. Weiss, Die Kristallstruktur von $SrAl_2$, *Z. Naturforsch.* **1969**, *24b*, 1191.

[2] S. Engel, E. C. J. Gießelmann, L. E. Schank, G. Heymann, K. Brix, R. Kautenburger, H. P. Beck, O. Janka, Theoretical and ^{27}Al NMR spectroscopic investigations of binary intermetallic alkaline-earth aluminides, *Inorg. Chem.* **2023**, *62*, 4260–4271.

[3] A. R. Schulze, H. Müller-Buschbaum, Zur Struktur von monoklinem $SrAl_2O_4$, *Z. Anorg. Allg. Chem.* **1981**, *475*, 205–210.

[4] S. E. Haszko, Intermediate Phases with the $MgCu_2$ Structure, *Trans. Metall. Soc. AIME* **1960**, *218*, 958.

[5] E. C. J. Gießelmann, S. Engel, S. Pohl, M. Briesenick, L. P. Rüthing, C. Kloos, A. Koldemir, L. Schumacher, J. Wiethölter, J. Schmedt auf der Günne, G. Kickelbick, O. Janka, Rapid synthesis of a green emitting phosphor by sulfidation of intermetallic $EuAl_2$ and its use in a hybrid material, *Chem. Mater.* **2025**, *37*, 97–108.

[6] K. Tezuka, Y. Tokuhara, M. Wakeshima, Y. J. Shan, H. Imoto, Y. Hinatsu, Crystal Structures and Properties of Europium Aluminum Oxynitride $Eu_2AlO_{3.75}N_{0.1}$ and Europium Aluminum Oxide $EuAl_2O_4$, *Inorg. Chem.* **2013**, *52*, 12972–12979.

[7] S. Geller, V. B. Bala, Crystallographic studies of perovskite-like compounds. II. Rare earth aluminates, *Acta Crystallogr.* **1956**, *9*, 1019–1025.

[8] U. Atzmony, E. R. Bauminger, I. Nowik, S. Ofer, J. H. Wernick, Isomer shifts and Mössbauer-effect efficiencies in intermetallic compounds of europium, *Phys. Rev.* **1967**, *156*, 262–266.

[9] H. H. Wickman, I. Nowik, J. H. Wernick, D. A. Shirley, R. B. Frankel, Hyperfine Fields and Isomer Shifts in Magnetically Ordered Europium Compounds, *J. Appl. Phys.* **1966**, *37*, 1246–1247.

[10] M. Nespolo, Charge distribution as a tool to investigate structural details. IV. A new route to heteroligand polyhedra, *Acta Crystallogr.* **2016**, *72*, 51–66.

[11] M. Nespolo, B. Guillot, CHARDI2015: charge distribution analysis of non-molecular structures, *J. Appl. Crystallogr.* **2016**, *49*, 317–321.
